# Study on the relationship between permeability coefficient and porosity, the confining and osmotic pressure of attapulgite-modified loess

**DOI:** 10.1038/s41598-023-43197-5

**Published:** 2023-09-26

**Authors:** Zhengrui Zhang, Ahad Amini Pishro, Lili Zhang, Xuejiao Ma, Siti Jahara Matlan, Nazaruddin Abdul Taha, Mojdeh Amini Pishro

**Affiliations:** 1 College of Civil Engineering, Sichuan University of Science & Engineering, Zigong, 643000 Sichuan People’s Republic of China; 2 Civil Engineering Programme, Faculty of Engineering, University of Malaysia Sabah, 88400 Kota Kinabalu , Sabah Malaysia; 3 School of Architecture and Design, Southwest Jiaotong University, Chengdu, 610031 China

**Keywords:** Chemical engineering, Civil engineering

## Abstract

This study investigated attapulgite-modified loess as an efficient and cost-effective method for creating an impermeable liner for landfills in regions with scarce clay resources. Laboratory permeability tests were conducted using a flexible wall permeameter to determine the permeability of compacted loess and attapulgite mixtures under varying osmotic conditions. The relationship between the permeability coefficient, attapulgite dosage, radial pressure, and osmotic pressure was analyzed. Nuclear magnetic resonance and scanning electron microscopy were also used to observe the microstructure of the modified loess. The results showed that attapulgite dosage significantly reduced the permeability coefficient, but the effect became limited when the content surpassed 10%. The decrease of the permeability coefficient of the modified loess is mainly due to the filling of pores between the loess by attapulgite, which makes the pore size and throat size of the modified loess smaller. The modified loess displayed a sheet structure that contributed to an increased permeability coefficient due to increased radial pressure. This study provides valuable insights into using attapulgite-modified loess as a material for landfill lining in regions with scarce clay resources.

## Introduction

In recent years, urbanization and advancements in living standards have led to a significant increase in domestic waste generation^1^. According to the China Urban Environmental Sanitation Association, the country's waste production is expected to reach 1 billion tons in 2020, with 400 million tons being household waste, 500 million tons being construction waste, and 10 million tons being kitchen waste^2^. The increase in waste generation poses a significant challenge regarding waste management, as improper disposal can result in environmental pollution and harm human health^3,4^.

Lanzhou City, located in Gansu Province, China, is on the Loess Plateau. It comprises six districts, including Qilihe, Anning, Chengguan, Honggu, Xingu, and "three counties and five districts" in Yongdeng County, Yuzhong County, and Gaolan County^5^. As of July 2020, the city's permanent population is estimated to be 4.134 million. The five districts surrounding the city produce approximately 4500 tons of waste daily. There are currently five unclosed and five urban landfills in the area, needing more anti-seepage layers, exhaust gas installations, and centralized treatment facilities for pipelines and leachate, resulting in inadequate waste treatment^6^. The soil in the area primarily consists of collapsible loess, which is a type of clay soil characterized by its yellow-brownish color, porosity of 40–50%, high water permeability, and strong chemical composition dominated by SiO_2_, followed by A1_2_O_3_, CaO, Fe_2_O_3_, and other minerals^7–9^. The topographical and geological conditions and the economic status of Lanzhou dictate that valley landfills should be prioritized in terms of construction, focusing on seepage prevention and slope stability.

Studies have been conducted on the effects of different materials on loess stabilization and seepage control. Silver nanoparticles (AgNPs) have been evaluated by Lee et al.^10^ for their ability to penetrate a clay liner in landfills. The results indicate that AgNPs cannot easily pass through the liner. Tian et al.^11^ studied the steady seepage in unsaturated loess soil. They found that it follows Darcy's law and that a new unsaturated soil permeameter has good performance and control. Other researchers include Li and Wang^12^, Wu et al.^13^, Liu et al.^14^, Song et al.^15^, Zhang et al.^16^, and Li et al.^17,18^ have studied the characteristics of loess under different conditions. Efforts to stabilize loess have included the use of chemical additives^19,20^, eco-materials^3,4, 21^, geopolymer^22^, and nano clay^23^. However, studies have yet to be conducted on the stabilization of loess for seepage control. Zhang et al.^24,25,26^ modified loess with lime to prevent seepage on a deep collapsible loess embankment slope, with the results indicating that the lime pile and anti-seepage wall had excellent performance, with the lime wall performing better than the lime-compacted pile. Bentonite clay is a commonly used material for loess stabilization. Zhang et al.^27^ researched the permeability of bentonite-modified loess as a landfill liner and established a regression model to predict its permeability.

The traditional methods for preventing seepage, such as the utilization of cohesive soil to enhance loess^28^, the implementation of bentonite composite geomembrane^27^, and others^29^, are not the ideal solutions for the local landfill in Lanzhou loess. This is because the availability of local bentonite in Lanzhou is limited, requiring transportation from other provinces^30^, which would increase the project cost. Cost control is crucial, especially in Lanzhou, an economically challenged region. Furthermore, the production cost of bentonite composite geomembrane is high. Any sharp objects, such as stones or branches in the slope, can easily damage the impermeable film during use, causing leachate to leak and pollute the environment^31^.

It can be seen from the above that bentonite, as a modified material, is widely used in landfill seepage prevention. Attapulgite and bentonite are a kind of special clay minerals, they have some similarities in chemical composition and some material properties. Attapulgite and bentonite have strong water absorption and moisture absorption properties, and can quickly absorb the surrounding water, forming a colloidal slurry. Both have relatively high specific surface area and porosity, giving them excellent performance in adsorption, adsorption of molecules and ions, etc. Due to their microstructure and porosity, both clay minerals can be used as waterproof and impermeable materials to some extent. Attapulgite and bentonite play an important role in many applications in the engineering field, such as soil improvement, waterproof materials, fillers, etc.

Attapulgite clay is abundant in Zhangye City in Gansu Province^32^. It is a type of cohesive soil formed in a Mediterranean to semi-arid climate and is mainly found in arid and semi-arid regions of the world^17,18^. It combines smectite and palygorskite^33^. Due to its high active silica and alumina content after calcination is widely used in the light industry, textiles, water treatment, and decolorization. Smectites are lattice clays that expand, with bentonite being a well-known generic name for smectite clays^34^. The palygorskite component is a needle-like crystalline form that does not swell or expand. Attapulgite creates gel structures in fresh and saltwater through a network of particles connected by hydrogen bonds^35,36^. The use of attapulgite in landfills is mainly for adsorbing^24,25^ and purifying leachate^37^. However, to date, no research studies the modification of collapsible loess with attapulgite for reducing permeability and using it as a landfill liner. Lanzhou, located in northwest China, has low rainfall and groundwater scarcity^38^. It is economically underdeveloped, making it necessary to prevent leachate from seeping into the soil and groundwater and endangering the residents' living and working conditions.

This study aims to modify local loess with local attapulgite and examine the relationship between the permeability coefficient, attapulgite addition levels, and different osmotic conditions. Nuclear magnetic resonance (NMR) and Scanning Electron Microscopy (SEM) were conducted to observe the microstructure of the attapulgite-modified loess. This study is of great significance in economically underdeveloped Lanzhou, where low rainfall and scarce groundwater threaten the environment if leachate seeps into the soil.

## Test materials and methods

### Test materials

This research utilized a specimen of loess collected from Lanzhou, China; the fundamental physical properties are listed in Table 1. The composition of the loess was evaluated through tests for grain composition, liquid limits, and plastic limits, as outlined in the "Standard for Geotechnical Test Methods" (GB/T 50123-2019). The analysis showed that the loess had an uneven grain size distribution and a high permeability due to large surface areas and pores in the soil particles. This finding is consistent with similar studies by Guo et al.^39^ and Song et al.^40^, who found that natural loess exhibits uniformity in soil structure and loose soil with a large pore structure.

**Table 1:** The basic physical properties of loess.

Particle size (mm)	> 1.1	1.1–0.25		0.25–0.075	0.075–0.05	0.05–0.01	0.01–0.005	< 0.005
Grain composition (%)	0.37		19.43	23.8	9.9	28.4	9.1	9.0

This study uses attapulgite, sourced from Zhangye City in Gansu Province, as a potential material for modifying local loess. The chemical composition of attapulgite is presented in Table 2, based on an attapulgite production inspection report. The data indicate that attapulgite contains high levels of S_i_O_2_ and N_a_OH, which can react with calcium ions in the loess to form calcium silicate, thus enhancing the soil's shear strength and reducing its permeability coefficient.

**Table 2:** The basic chemical composition of attapulgite.

Composition	SiO_2_	AL_2_O_3_	Fe_2_O_3_	Na_2_O	K_2_O	Cao	MgO	MnO	TiO_2_	NaOH
Percentage (%)	60.5	10.1	6.7	0.11	1.3	1.95	11.35	0.61	0.63	11.8

Research conducted by Song et al.^40^ investigated the fractal characteristics of the particle size distribution (PSD) of various minerals in loess and loess treated with lime. They found that fine particles in the lime-treated loess were well connected, causing the soil structure to change from loose to dense, which is attributed to the high content of S_i_O_2_ in lime, which significantly increased the soil's shear strength.

### Compaction test

The air-dried loess specimen was mixed with various amounts of attapulgite (2%, 4%, 6%, 8%, 10%, and 16%) in the appropriate proportions and thoroughly stirred to ensure even distribution. Water was sprayed, and the soil specimen was mixed by stirring based on water content measurements. The prepared specimens were stored in sealed plastic bags and tested for compaction after 48 h using the JDS-2 electric standard light compaction instrument. The compaction cylinder had an inner diameter of 102 mm, a volume of 947.4 cm^3^, and a 2.5 kg hammer with a 51 mm bottom diameter. The drop was 305 mm, delivering 592.2 kJ/m of energy. The compaction test method followed the ASTM D698 Standard, consisting of three layers with 25 strokes. The residual soil height should be at most 5 cm. Table 3 shows the compaction test results.

**Table 3:** Compaction test results.

Loess soil (%)	Attapulgite dosage, R_A_ (%)	Optimum moisture content (%)	Maximum dry density (g/cm^3^)
100	0	13.5	1.88
98	2	13.9	1.85
96	4	13.23	1.84
94	6	16.52	1.74
92	8	17.01	1.71
90	10	17.3	1.70
84	16	18.85	1.69
0	100	40	1.26

### Permeability, NMR, and SEM tests

When the permeability coefficient of a porous material is 1.0 × 10^–7^ cm/s or less, the lateral wall leakage of a traditional rigid wall permeameter can significantly affect the accuracy of the permeability coefficient measurement. The GDS-PERM flexible wall permeameter, which offers accurate measurement and pressure penetration, was used in this test to determine the permeability coefficient of the modified loess under constant head conditions. The GDS Company produces this permeameter in the UK, which conforms to the ASTM D5084-03 standard. The modified loess's permeability coefficient was measured using a constant head method.

**Figure 1:**
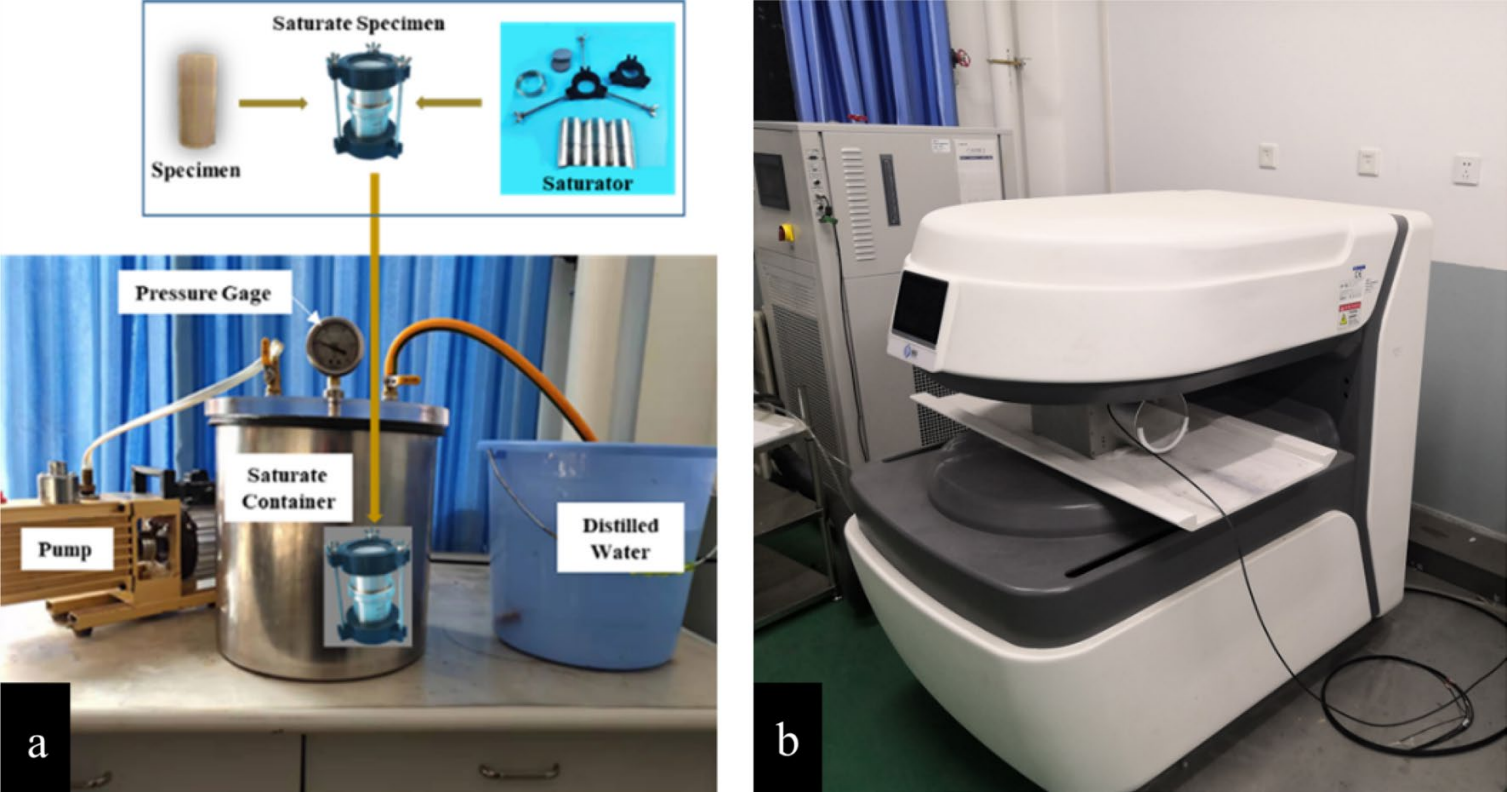
NMR test picture, (**a**), Sample vacuum saturation, (**b**), Porosity detection.

In the test, distilled water was used as the percolation fluid, and each material's maximum dry-density compaction specimen was selected as the penetration specimen. The specimen diameter was 100 mm, and the height was 50 mm. Before the penetration test, radial pressure was set at 100 kPa. Backpressure of 30 kPa, 60 kPa, and 90 kPa was applied step by step to saturate for 1, 3, and 5 days to ensure the specimen reached full saturation. During the seepage process, distilled water enters from the top of the specimen and flows out from the bottom. The air compressor provided radial, base, and back pressures and was applied to the specimen through the gas–liquid interface. After the test, the permeability coefficient of the modified loess with different attapulgite content could be obtained according to the calculation method of Darcy's law's constant head permeability coefficient. After the permeation test, the saturated specimen was removed and put into the nuclear magnetic resonance apparatus to conduct the NMR test. Before and after the permeation test, the specimen was collected from the permeation specimen, which would be used for the SEM test. Figure 1 is a picture of testing the moisture content of a soil specimen. Water content was measured by NMR (MacroMR12-150H-1) produced in Jiangsu, China. The principle of this method to test the water content is to detect the hydrogen atoms of water molecules between the pores inside the specimen and then convert it into the water content, so before the test, we must ensure that the specimen is fully saturated. Otherwise, the test results will have errors. Figure 1a shows the specimen saturation process, which adopts the vacuum saturation method, and the test meets the requirements of ASTM D4643-16. After the specimen with the belt is fully saturated, the specimen is taken out for a porosity test, as shown in Fig. 1b. Through this test, not only the moisture content of the specimen can be obtained, and the size distribution of the internal pore diameter and the pore throat of the specimen can be obtained.

## Result and discussion

### Relationship between the optimum water content, maximum dry density, and addition rate of attapulgite

**Figure 2:**
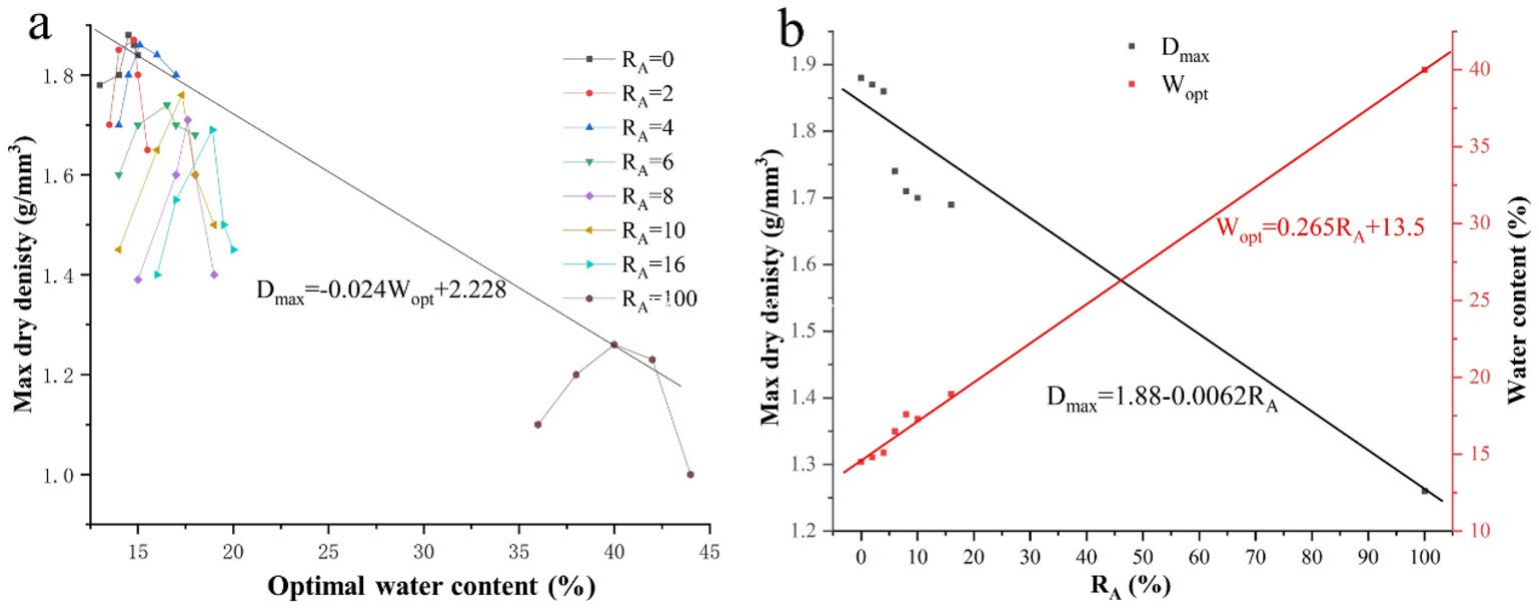
Compaction test results about modified loess with different percentages of attapulgite.

This experiment performed the compaction test of specimens with different attapulgite content. The experimental results obtained the relationship between the maximum dry density, the optimum moisture content, and the attapulgite-modified loess content. From Fig. 2a, we can see that the maximum dry density of the specimen decreased with the increase of attapulgite content. On the contrary, the optimal water content increased. This is because the density of attapulgite is lower than loess, and the increase in its percentage in the specimen will inevitably reduce the dry density of the specimen. Compared with loess, attapulgite has more clay particles and a smaller particle volume, which means more surface area, so it needs more water when mixing them. In addition, the silicon and calcium ions in the two soils react to form substances such as silicic acid. This process also requires water. All the above reasons lead to the optimum moisture content of attapulgite-modified loess increasing with the increase of attapulgite content^21,23,40^.

It can also be seen from Fig. 2a that the relationship between the optimal water content and the maximum dry density varies linearly. Therefore, the relationship between the optimal water content's maximum dry density and the attapulgite dosage can be obtained through Fig. 2b.

1 $${\mathrm{D}}_{max}={-0.024\mathrm{W}}_{OPT}+2.228$$

2 $${\mathrm{D}}_{max}=1.88-0.0062{\mathrm{R}}_{A}$$

3 $${\mathrm{W}}_{opt}=0.265{\mathrm{R}}_{A}+13.5$$

Equation (1) describes the relationship between D_max_ and W_opt_; Eq. (2) describes the relationship between D_max_ and R_A_, and Eq. (3) describes the relationship between W_opt_ and R_A_, where D_max_ represents the maximum dry density (g/mm^3^); W_opt_ represents the optimal water content (%); R_A_ represents the dosage ratio of attapulgite (%).

### Permeability performance

**Figure 3:**
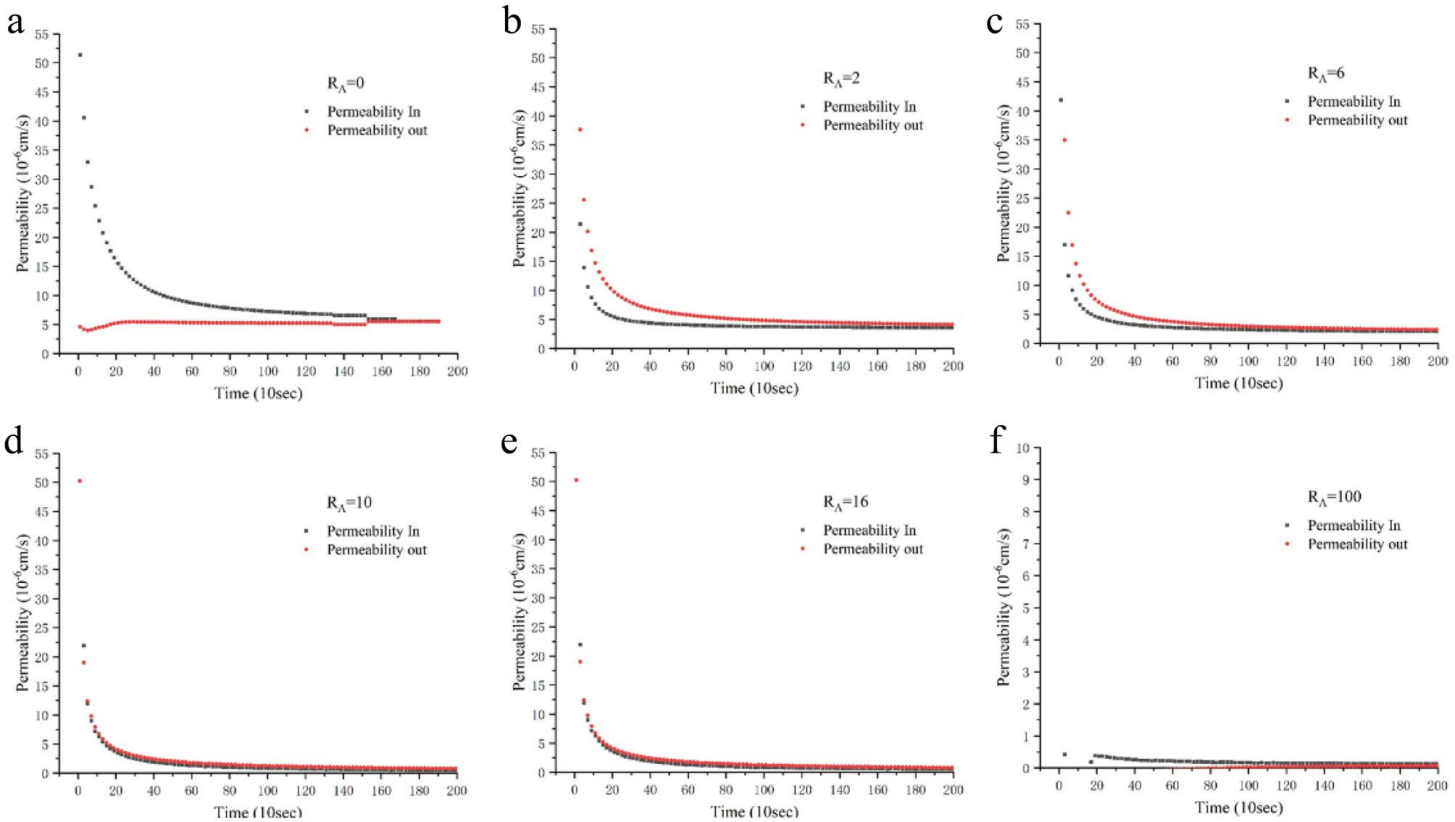
Osmotic test results of attapulgite modified loess.

In the osmotic experiment, the criterion for the end time of the test is that the values of permeability in (i.e., inflow) and permeability out (i.e., outflow) are nearly equal, or the two data remain unchanged, and their average value is taken as the result of the test. If the data can get through the software in time, set the frequency to once every 10 s. Figure 3 shows the test result of attapulgite-modified loess with a radial pressure of 100 kPa and osmotic pressure of 20 kPa. R_A_ represents the dosage ratio of attapulgite (percent, %). As can be seen from the figures, except for Fig. 3a, the inflow value is always greater than that of the outflow. In the pure loess specimen test, the water was set to permeate from bottom to top, so the value of Permeability In was less than that of Permeability Out. With the water migration inside the soil specimen, the water yield at the bottom gradually gets close to the water intake at the top.

Currently, the two permeability coefficients measured by the sensors at the top and bottom are nearly equal; with increased attapulgite content, the permeability coefficient decreases. In the experiment, the specimens with higher attapulgite content took longer to stabilize the permeability. There are very few cases where inflow and outflow are precisely equal. Often, they are just infinite approximations, and the rate of change is slower and slower. In particular, the specimens' permeability coefficient with a high attapulgite content was initially low. In the later stages of the experiment, the data kept changing, even after three days of the experiment. However, the change is less than e-12, which has little effect on the experiment's results, so we choose to end the experiment and use the average value as the final permeability coefficient. Figure 3 is just a partial data capture in the first few hours of the experiment.

**Figure 4:**
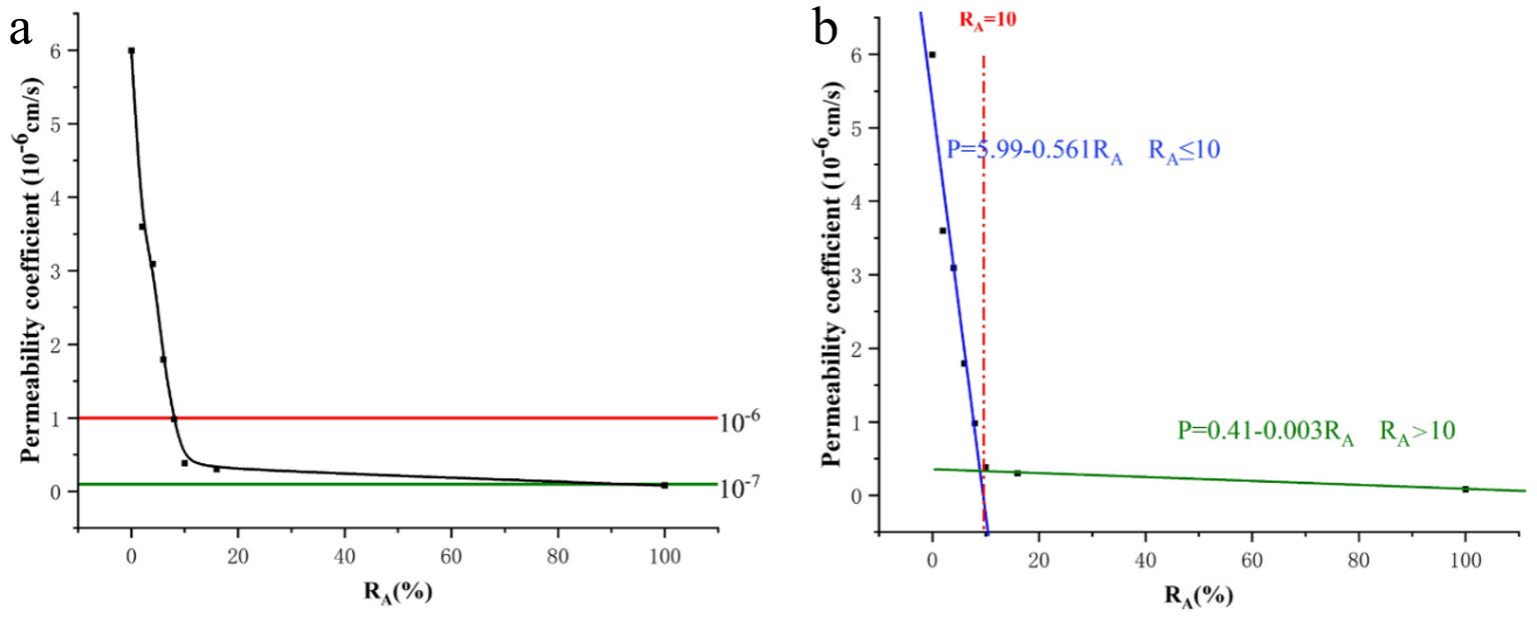
Relationship between the permeability coefficient and addition rate of attapulgite.

The final test results are shown in Fig. 4. From Fig. 4a, we can see that the permeability coefficient decreases with the increase of the attapulgite ratio. When the attapulgite content is less than or equal to 10%, the decreasing rate is significant, and the relationship is linear. The permeability coefficient of loess changed from 5.99 × 10^–6^ to 0.38 × 10^–6^ cm/s after adding 10% attapulgite, which is a nearly 15-fold reduction. These results are consistent with those of bentonite-modified loess by Zhang et al.^27^ and lime-modified loess anti-seepage research by Zhang et al.^24,25^. However, when the attapulgite content exceeds 10%, the permeability coefficient reduction rate decreases slowly. From 10 to 16%, the permeability coefficient decreased by 0.08 × 10^–6^ cm/s, and, with the increase in the attapulgite content, the curve became flat. This shows that the attapulgite can improve the permeability coefficient of loess up to its maximum value after the content of attapulgite reaches 16%. Referring to the national code of GB16889-2008, the permeability coefficient of the impermeable landfill layer should not be greater than 1 × 10^–7^ cm/s. The study's results suggest that adding attapulgite can significantly improve the permeability performance of loess. However, the amount of attapulgite required to meet the specified permeability coefficient in engineering codes is relatively high, at approximately 80%. While this amount may seem excessive, the improvement in permeability performance makes attapulgite-modified loess a viable alternative material for use in engineering projects, such as landfill liners. Attapulgite is used to modify loess mainly to play the adsorption performance of attapulgite and provide a second safety barrier for landfill. The results show that the attapulgite can reduce the permeability coefficient of loess, which provides a prerequisite for the feasibility of the anti-seepage lining material with adsorption.

Since the two curves before and after the dosage of 10 are nearly linear (Fig. 4b), the relationship between the permeability coefficient and the addition rate of attapulgite can be expressed by the following formulas (4).

4 $$\mathrm{P}\left({\mathrm{R}}_{A}\right)=\left\{\begin{array}{c}5.99-0.561{\mathrm{R}}_{A} {\mathrm{R}}_{A}\le 10\\ 0.41-0.003{\mathrm{R}}_{A} {\mathrm{R}}_{A}>10\end{array}\right.$$

where P represents the permeability coefficient (10^–6^ cm/s), ${\mathrm{R}}_{A}$ represents the dosage of attapulgite (%). The formula can predict the relationship between the permeability coefficient of modified loess and attapulgite content. The permeability coefficient of modified loess with different attapulgite content can be obtained.

The relationship between the permeability coefficient and other variables can be complex, and the nonlinear relationship between the data can be more accurately captured by using curves for fitting. Curves can be adapted flexibly to various data distributions to provide better fitting results, such as polynomial, exponential, logarithmic, etc. However, in some specific cases, such as this study, the relationship between the permeability coefficient and other variables is linear, in which case a straight line can be used for fitting. Gale^41^ used the linear fitting method when studying the soil permeability coefficient. Huisman and Feddes^42^ studied the permeability coefficient of soil water movement and adopted the linear fitting method. Kandelous^43^ used the linear fitting method when investigating the permeability coefficient of water in soil. Linear fitting is the simplest method suitable for data sets with obvious linear relationships.

**Figure 5:**
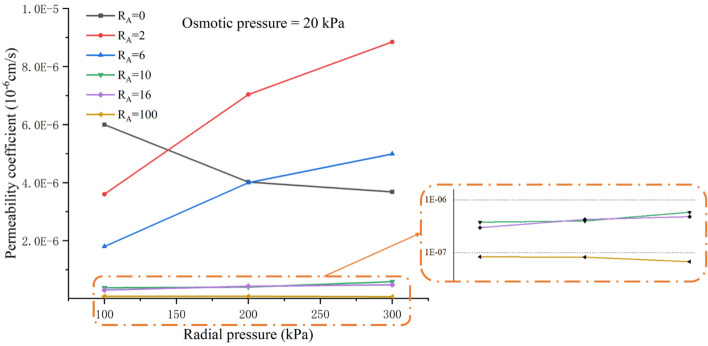
Relationship between the permeability coefficient and radial pressure.

Figure 5 shows the influence of constant osmotic pressure and a stepwise increase in radial pressure on the permeability coefficient. It can be seen from the figure that the permeability coefficient of the pure loess specimen decreases with the increase of radial pressure. The permeability coefficient of pure attapulgite also decreases with increased radial pressure. Still, the decrease is much smaller than the loess trend with increased radial pressure. On the contrary, the permeability coefficient of the attapulgite-modified loess increases with the increase of radial pressure. According to previous research on the permeability coefficient of granular soil, as the cell pressure rises, the surrounding pressure squeezes the soil, the soil volume decreases, and the pores within the soil shrink, resulting in a decrease in the permeability coefficient^27^. The test results of the loess specimens in this test conformed with this rule. In the analysis of the SEM test results later in this paper, the opposite result of attapulgite modified loess will be explained.

**Figure 6:**
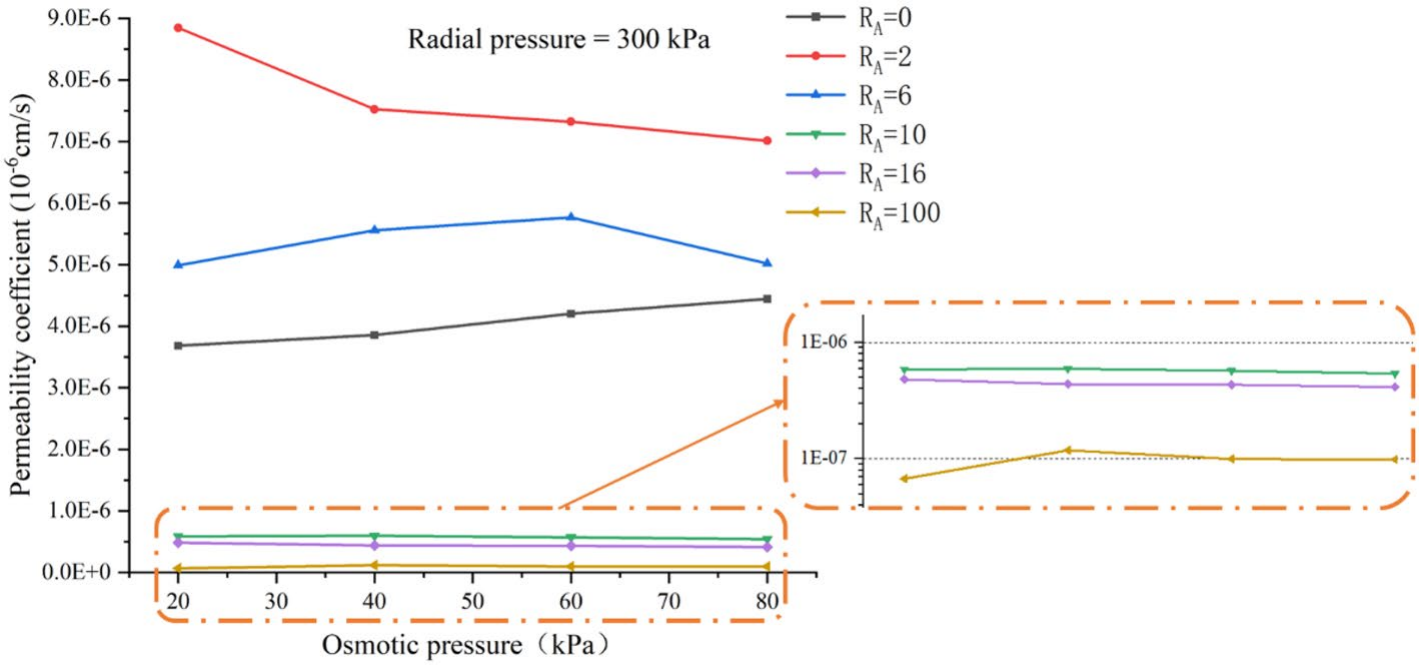
Relationship between the permeability coefficient and osmotic pressure.

Figure 6 depicts the effect of constant radial pressure and stepwise osmotic pressure increases on the permeability coefficient. Since this test was carried out after the constant osmotic pressure and the change of radial pressure, the permeability coefficient of the loess decreased under the influence of the increased radial pressure. Therefore, at the beginning of this test, the permeability coefficient of the loess was lower than that of the attapulgite-modified loess. In the experiment, the radial pressure was kept constant at 300 kPa, and the osmotic pressure increased from 20 to 80 kPa step by step. It can be seen from the interpretation results that the permeability coefficient of the loess increased from 3.68 × 10^–6^ to 3.86 × 10^–6^ cm/s with the osmotic pressure increasing from 20 to 80 kPa. According to Darcy's law, the permeability coefficient is directly proportional to the height of the water head. Increasing the osmotic pressure is equivalent to increasing the height of the water head, so the permeability coefficient will inevitably increase. Since loess is a granular soil with a relatively developed pore structure, the water infiltration rate into the pores will accelerate when the osmotic pressure increases, and then the permeability coefficient increase. However, the permeability coefficient showed a decreasing trend for the attapulgite-modified loess. For attapulgite modified loess, the results are contrary. Zhang et al.^27^ and Song et al.^40^ conducted permeability tests on bentonite-modified loess and found similar findings to this experiment. As a result, it can be concluded that as osmotic pressure rises, the permeability coefficient of loess modified by fine clay decreases. This is because, under the influence of water flow, the fine particles in the specimen migrate along the direction of water permeability. Under osmotic action, the attapulgite unbound to the loess migrates from top to bottom in this experiment, eventually settling at the bottom of the specimen. This action prevents the liquid from penetrating the bottom, lowering the permeability coefficient. Later in this article, SEM tests will be used to confirm this result.

### NMR test results

**Figure 7:**
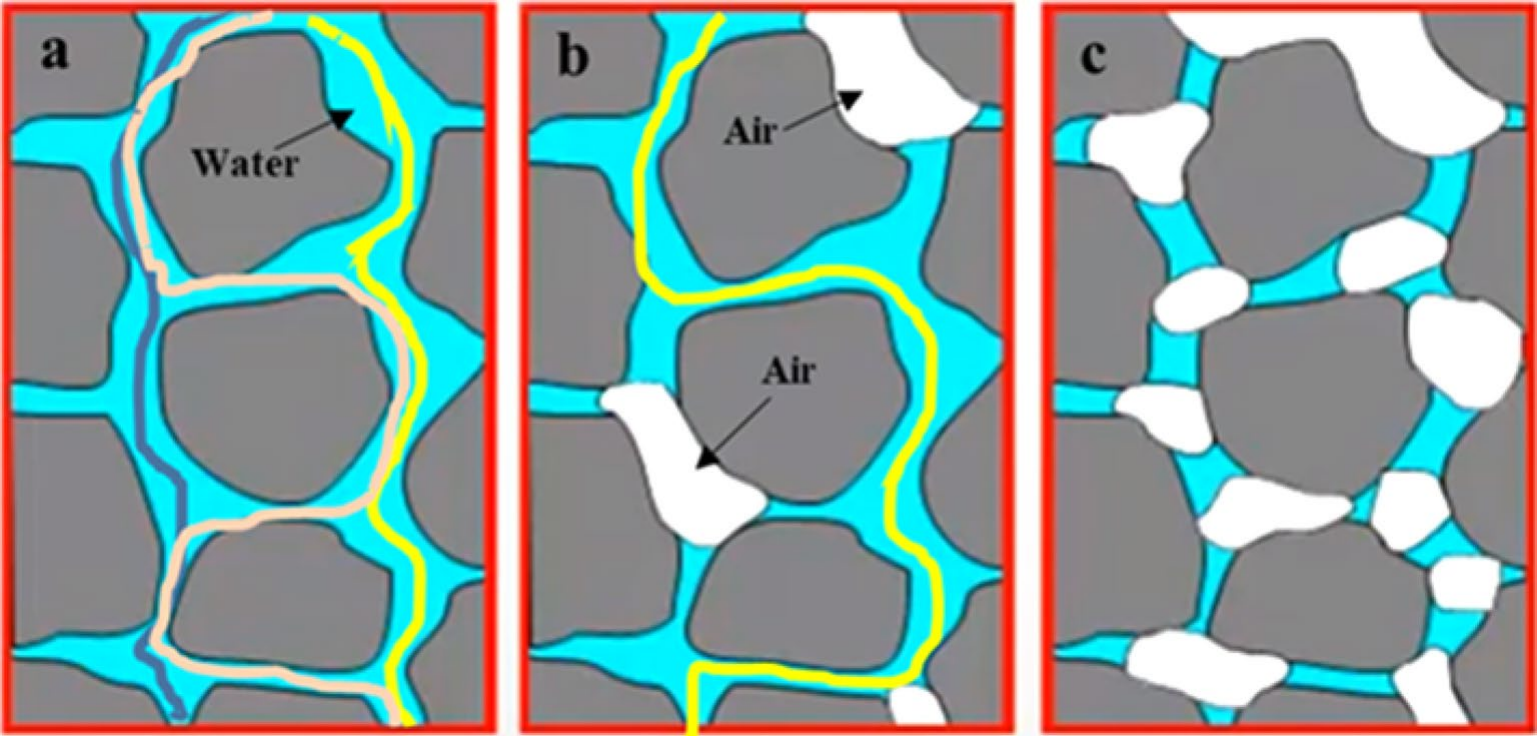
Schematic diagram of the effect of air on permeability coefficient.

The degree of compactness, particle size, and internal porosity of the sample will affect the permeability coefficient of the sample. Still, even under the same spatial structure, the air content in the pore will also affect the seepage path of water and thus affect the permeability coefficient of the soil sample. Figure 7 is the schematic diagram of the effect of air on the permeability coefficient; Fig. 7a–c shows the influence of air on the permeability coefficient under three different internal structures. In the picture, the blue represents water, and the white represents air. Figure 7 is one of the largest; three groups of permeability inside can be seen from the diagram, the seepage channels were all filled with water to fill in a sample, there is no air, dilute flow is continuous, and the particle pore is Unicom before, this makes the who can more easily in channel air circulation, and the seepage path from top to bottom there are many articles, can be along the left side, as shown in dark blue lines, It can also be percolated along the right side, as shown by the yellow lines, or it can be percolated along an S-shaped path from the middle. The permeability coefficient of the sample is the highest. As shown in Fig. 7b, compared with Fig. 7a, three air sacs of different sizes appear in the seepage path. Due to the existence of air sacs, water cannot pass through the middle of the air sacs, making the seepage path of water bypass the air sacs. Finally, there is only one path for the water flow to flow quickly, as shown in the loess line in the Figure. Therefore, the seepage difficulty of this sample is more incredible than sample a's, and the permeability coefficient is more minor than sample a. There are more airbags in Fig. 7c than in Fig. 7b. The airbags form many obstacles in the path of water flow seepage, and finally, the water flow cannot pass through the sample. Therefore, the permeability coefficient of this sample is the smallest among the three groups of samples. For the sample with significant air content, as shown in Fig. 7c, natural saturation could be better because it is difficult for the water pressure or capillary opposing force to discharge the air inside, and the sample will not be completely saturated. Therefore, to thoroughly saturate the sample, we must adopt the vacuum saturation method mentioned above by pumping out all the air inside the sample so that the moisture can fill the internal pores. According to the interpretation and analysis in Fig. 7, we can see that the gas content of the sample will affect the water content, and the water content directly reflects the permeability coefficient of the sample. Therefore, water content, permeability coefficient, and matric suction are closely related.

The accuracy of NMR experimental results largely depends on the saturation of the specimen, which is typically determined based on the image of the hydrogen nucleus in water. Accurate porosity results can be obtained for fully saturated specimens since the pores are filled with water. In this experiment, the specimen was first saturated before penetration testing and assumed to be saturated throughout the test. After the osmotic experiment, the specimen was removed from the permeameter and subjected to NMR testing to obtain its pore distribution results.

**Figure 8:**
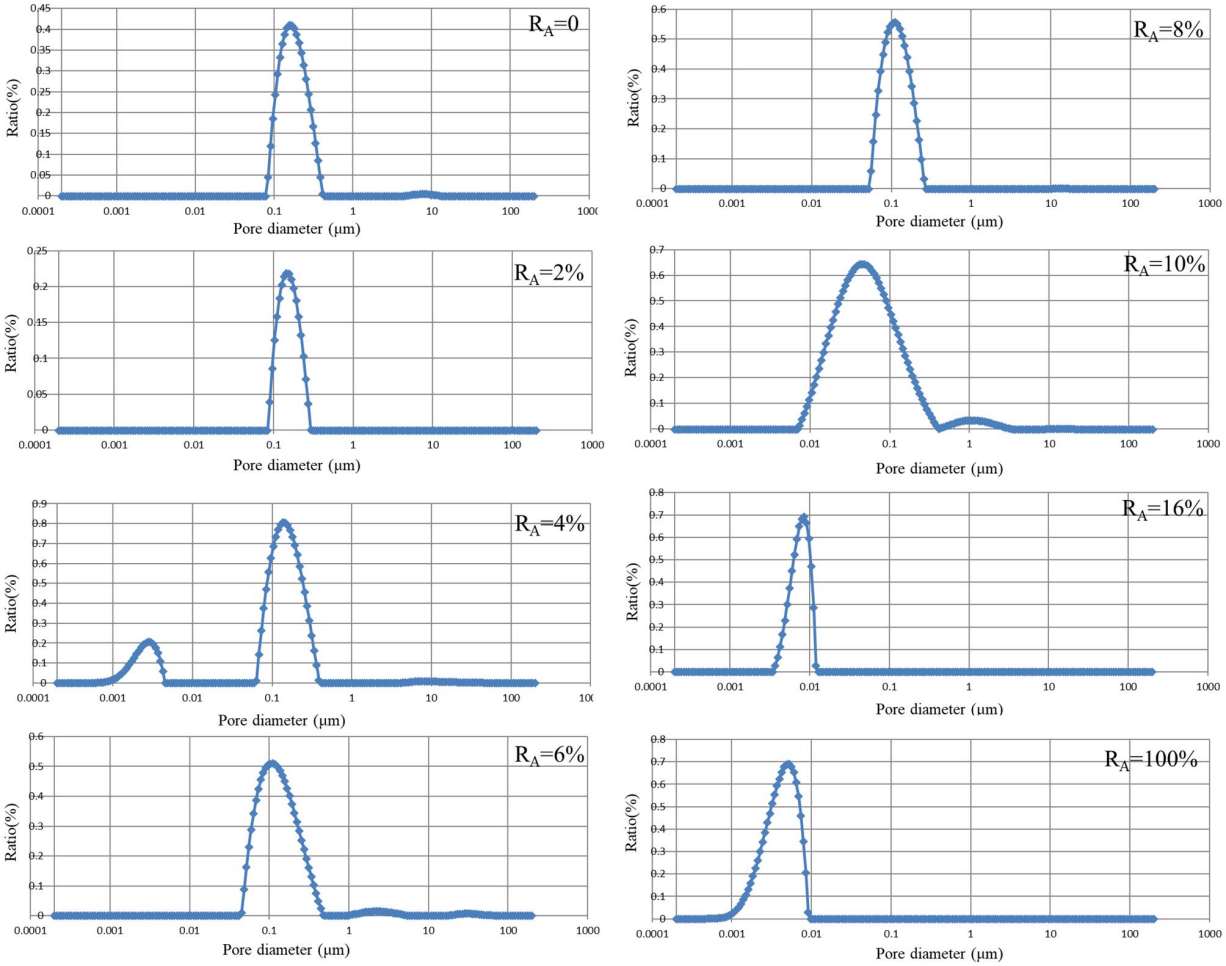
NMR test results for modified loess with different percentages of attapulgite.

The distribution of pores with varying diameters in the specimen under different attapulgite dosages is shown in Fig. 8. The figure reveals that the loess's pore size is predominantly distributed within 0.1–0.8 μm. As the attapulgite content increases, the proportion of more significant pore sizes decreases, and the peak of the curve shifts towards the left. At an attapulgite content of 100%, the pore size distribution is primarily concentrated between 0.001 and 0.01 μm. The test outcomes indicate that as the attapulgite content increases, the large pores in natural loess are filled with attapulgite, which causes a decrease in the soil porosity and permeability coefficient. In a study by Yang et al.^44^, the researchers examined bentonite-modified loess using SEM and observed that the pore diameter decreased as the bentonite content increased. This finding is comparable to the results of our investigation.

**Figure 9:**
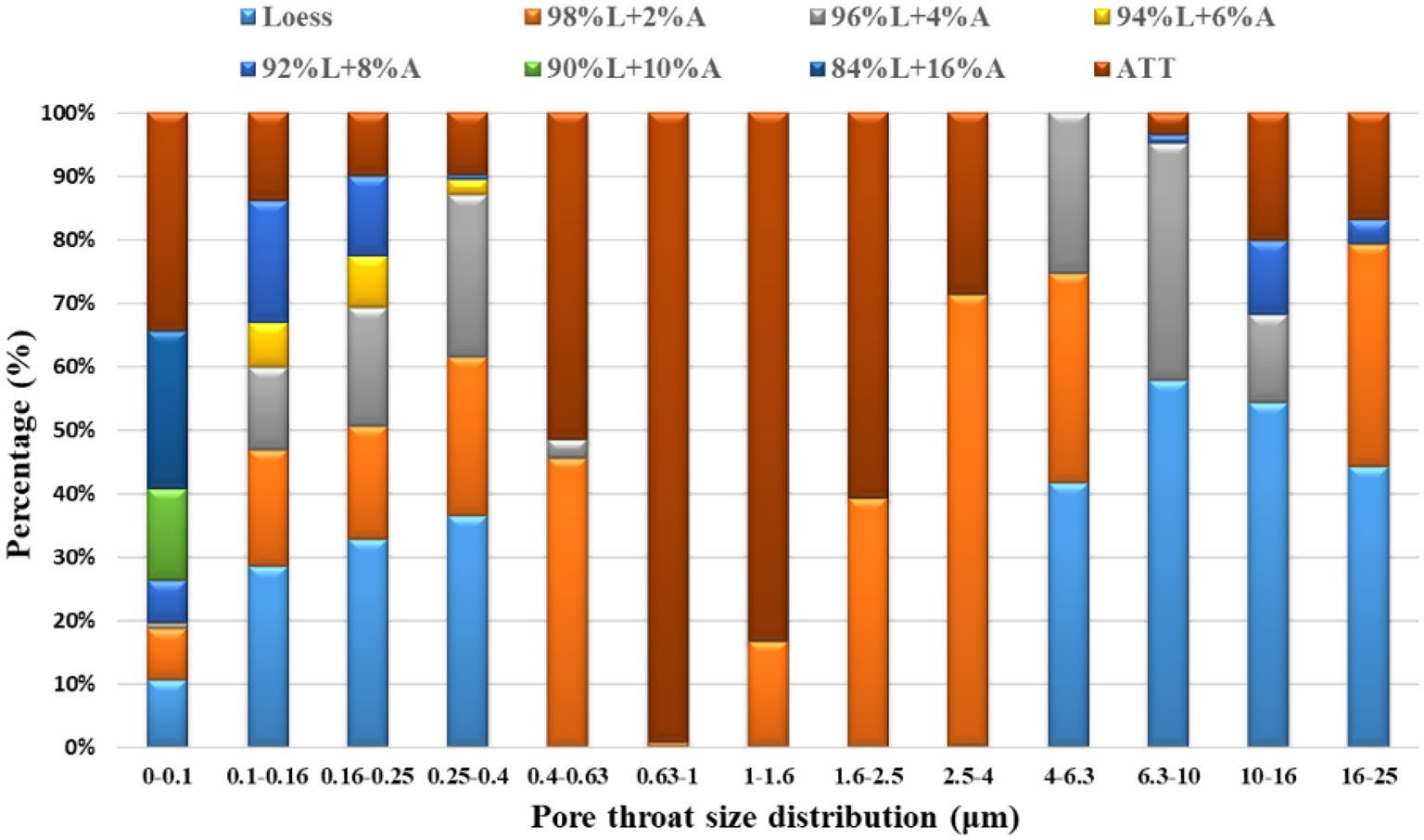
Pore throat size distribution of modified loess with different percentages of attapulgite.

Figure 9 shows the pore throat size distribution of attapulgite-modified loess at saturation. As can be seen from the public figure, similar to the distribution of pore size in Fig. 7, the proportion of small-size pore throats increases with the increase of attapulgite content, while on the contrary, there are more large-size pore throats in loess. The pore diameter throat is the critical factor that directly affects the permeability coefficient of the soil sample. The reduction of the pore size of the throat makes it more difficult for water to permeate, thus reducing the permeability coefficient. It can be concluded that including attapulgite can effectively reduce the size of the pore throat of modified loess and improve its impermeability.

**Figure 10:**
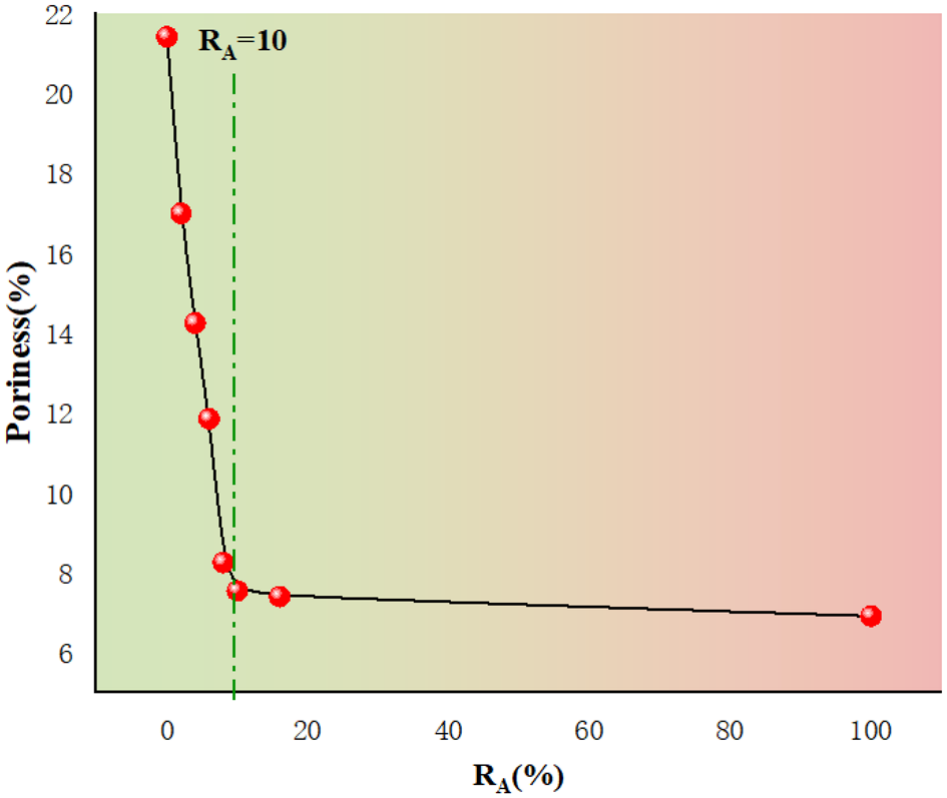
Relationship between the change of pore and addition rate of attapulgite.

The test results show that increasing attapulgite content leads to a significant decrease in pore diameter and the total number of pores, indicating the increased density of the specimen and a decrease in permeability coefficient. The increase in attapulgite content causes a significant decrease in pore diameter and number, indicating an increase in specimen density and a decrease in the permeability coefficient. Figure 7 shows that at a 10% dosage of attapulgite, the pore diameter distribution ranges from 0.08 to 0.4 μm, and the proportion of pores with a diameter of 0.1 μm is 56%. The porosity of the specimen with attapulgite content of 6% and 16% was determined to be 7.5% and 7.4%, respectively, indicating little difference in permeability coefficient between the two specimens. According to the data of the permeability test, their permeability coefficients are 0.38 × 10^–6^ cm/s and 0.30 × 10^–6^ cm/s, respectively. Therefore, the permeability test is consistent with the NMR test results. Figure 10 shows that as the attapulgite content increases, the porosity of the specimen decreases. The reduction rate is very significant when the attapulgite content is less than 10%, but for dosages more significant than 10%, the rate decreases slowly, and the curve becomes flat. This variation pattern is very close to the permeability coefficient in Fig. 4, proving that porosity is a subjective factor directly affecting the permeability coefficient.

It can be seen from the figure that 10% is a critical value, before which the permeability coefficient's decline rate is very fast, and after exceeding 10%, the rate drops rapidly. The reason for this is the change in porosity. The void of undisturbed loess is large, and the porosity and permeability of loess decrease after adding attapulgite. With the increase of attapulgite content, some large pores in loess are filled. The higher the content of attapulgite, the lower the porosity of loess. However, when the content reaches 10%, some easily filled pores are almost completely filled, so the decline rate is fast before 10%. After more than 10%, the remaining tiny pores are not easily filled, so the rate of decline slows down. Therefore, porosity is a direct factor affecting the permeability coefficient.

### SEM test results

**Figure 11:**
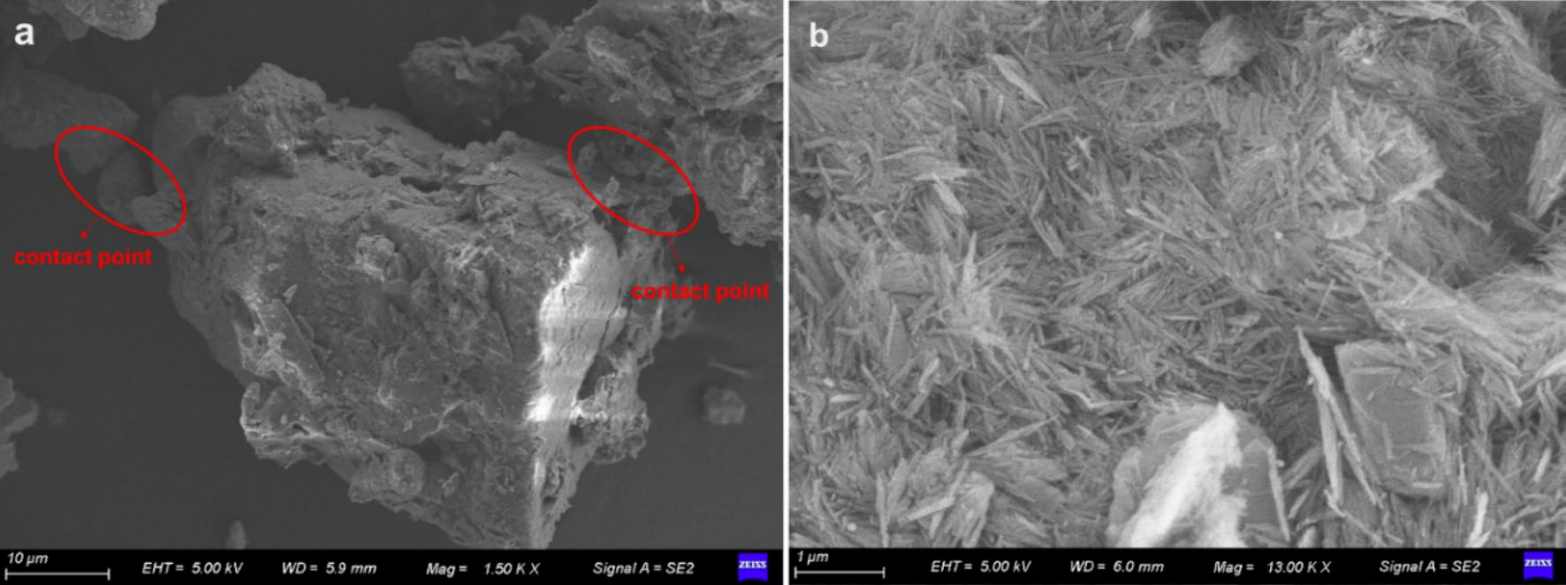
Microstructure of the materials, (**a**). loess; (**b**). attapulgite.

Figure 11 is the SEM picture of loess and attapulgite. When attapulgite is added to the loess, the particles of attapulgite can fill the pores between the loess particles, as seen in Fig. 11b. Combining the loess and attapulgite results in a more compact structure, as shown in Fig. 11b. The structure of the soil becomes denser, which can increase the shear strength of the soil and reduce the coefficient of permeability. These research findings agree with the conclusions drawn by Song and colleagues, who observed that adding lime to loess could enhance soil structure and increase the soil's shear strength^44^. In a dry state, soil particles are held together by inter-particle connections that confer a certain degree of strength to the soil. However, when the soil is soaked, these connections can dissolve, leading to the movement of particles and ultimately causing subsidence (Fig. 11a). The subsidence can damage buildings, infrastructure, and other adverse environmental effects. The microstructure of attapulgite and loess can be observed in Fig. 11b, which shows that attapulgite has a fibrous or needle-like structure and a lower porosity than loess. These results explained why adding attapulgite decreases porosity and permeability coefficient with increasing attapulgite content. These findings are consistent with the results of a previous study by Song et al.^40^. Attapulgite's needle-like structure gives it a large surface area, which makes it useful in various industrial applications, including adsorption, catalysis, and as a reinforcing filler in composite materials^32^.

**Figure 12:**
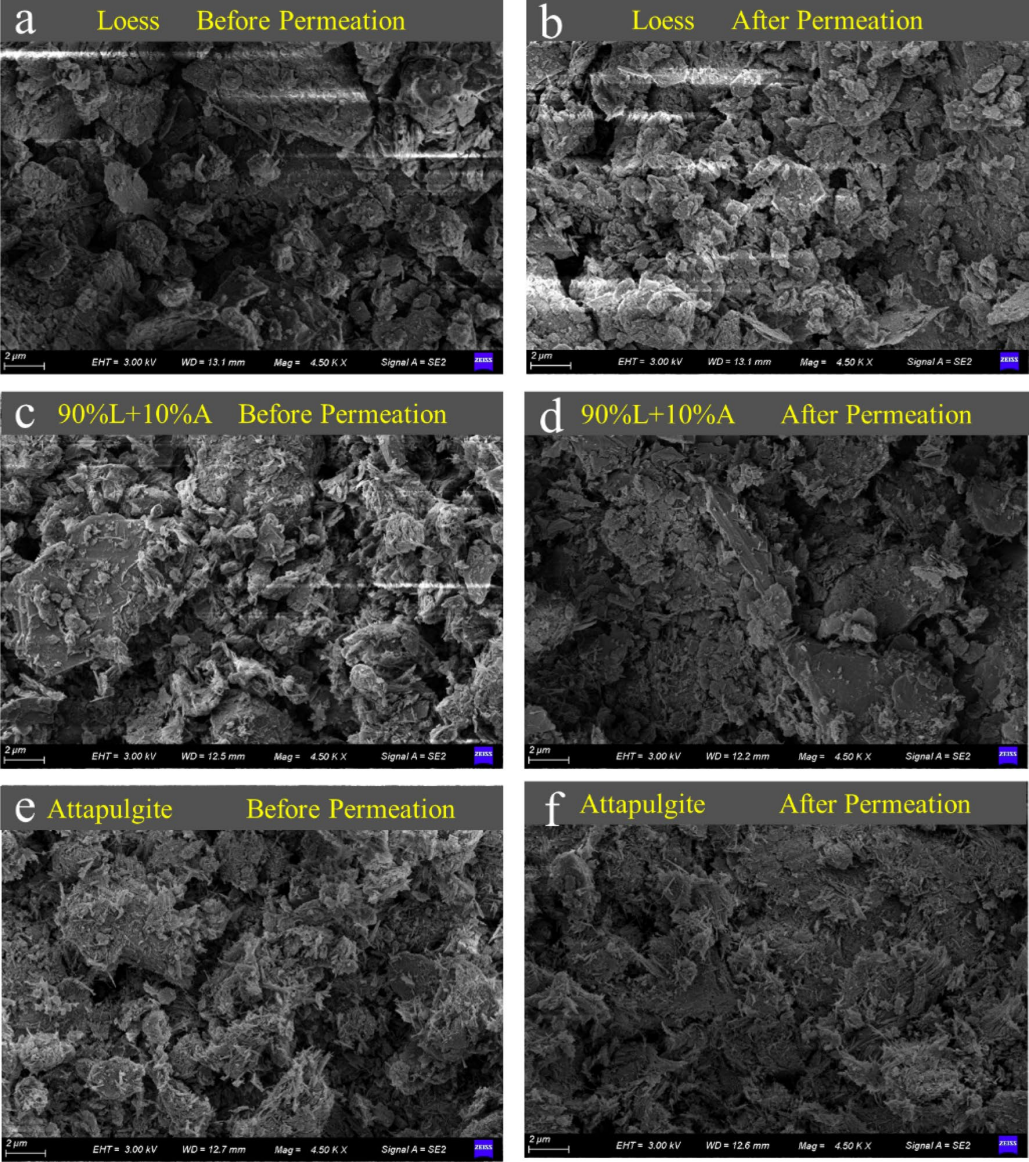
Microstructure of the loess, attapulgite modified loess, and attapulgite.

Figure 12 depicts SEM images of loess, attapulgite-modified loess, and pure attapulgite specimens before and after penetration. Figure 12a displays the SEM image of loess before infiltration, indicating that the specimen has a high porosity, with dots connecting the particles. Although this observation is similar to Fig. 11a, which examines undisturbed loess, the pores appear more extensive in the latter. Notably, Fig. 12a represents the SEM image of disturbed loess. After compacting soil during specimen preparation, its spatial structure becomes denser than natural loess. Figure 12b depicts an SEM image of the loess specimens after infiltration, indicating that the intergranular pore of the loess decreases, and the soil becomes more compact. This decrease in pore size is consistent with previous studies by Zhang et al.^24,25^, Yang et al.^44^, and Guo et al.^39^, all of which observed a reduction in the collapsibility of loess. When collapsible loess is exposed to water, the cementing material between the particles dissolves, causing the particles to move downwards due to gravity. As a result, the soil reaches a state of stability and appears to have subsided.

Figure 12c,d show SEM photos of attapulgite-modified loess before and after infiltration. The attapulgite-modified loess in Fig. 12c appears to have a flake structure, with attapulgite-modified loess particles linked together to form larger flakes. This structure reduces the modified loess's porosity and decreases its permeability coefficient. Figure 12d shows how the spatial structure of the modified loess specimen changed after infiltration tests were carried out under different cell pressures (100 kPa, 200 kPa, and 300 kPa). The original horizontally arranged sheet structure changed into a vertically warped structure, with the sheet structure compressed against each other to form more significant gaps. This spatial structure change can explain the increased permeability coefficient of the modified loess with the increase in cell pressure during the permeability test. The findings of Yang et al.^45^ on the improvement of loess with modified polypropylene fiber and cement are comparable to those of this study.

Figure 12e,f show the SEM photos of pure attapulgite specimens before and after infiltration. As can be seen from Fig. 12e, the flocculent lamellar structure is formed by the extrusion of the jack during specimen preparation, and the structure becomes denser after infiltration. The permeability coefficient of the attapulgite specimen is the most minor compared with loess and modified loess, and the permeability coefficient tends to decrease with the increase of radial pressure (Fig. 12f). This is consistent with the conclusion of the previous permeability test.

**Figure 13:**
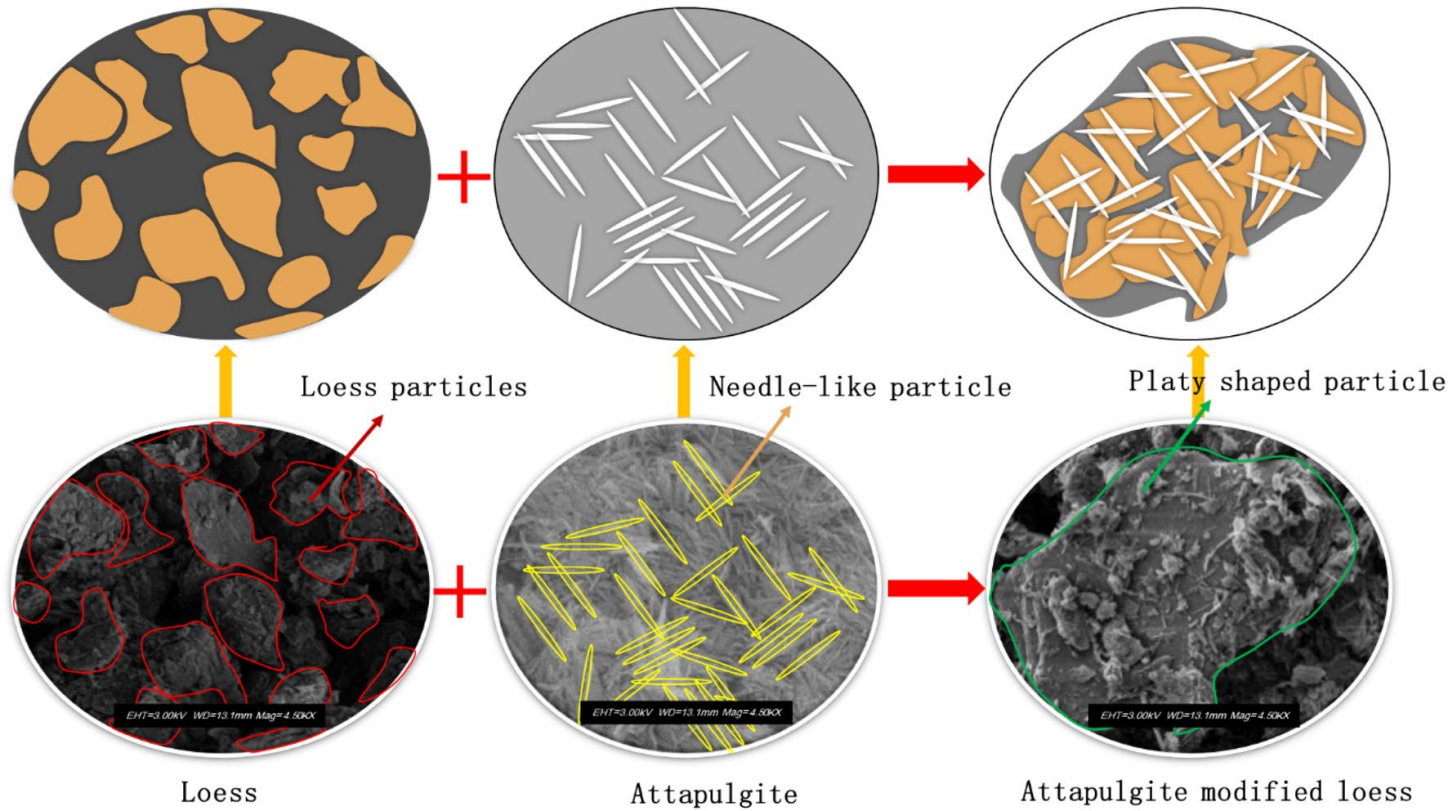
Schematic diagram of the microstructure changes of attapulgite modified loess.

Figure 13 depicts the change process in the spatial structure of attapulgite modified loess. Attapulgite shows a needle-like structure with a minor porosity. After mixing loess and attapulgite, the loess particles combine with the needle-like particles of attapulgite to form a large-volume sheet structure. In this process, attapulgite acts as a reinforcing bar, connecting the loose loess particles to form a larger sheet structure with a lower porosity than the initial state of the loess and, thus, a significantly lower permeability coefficient.

**Figure 14:**
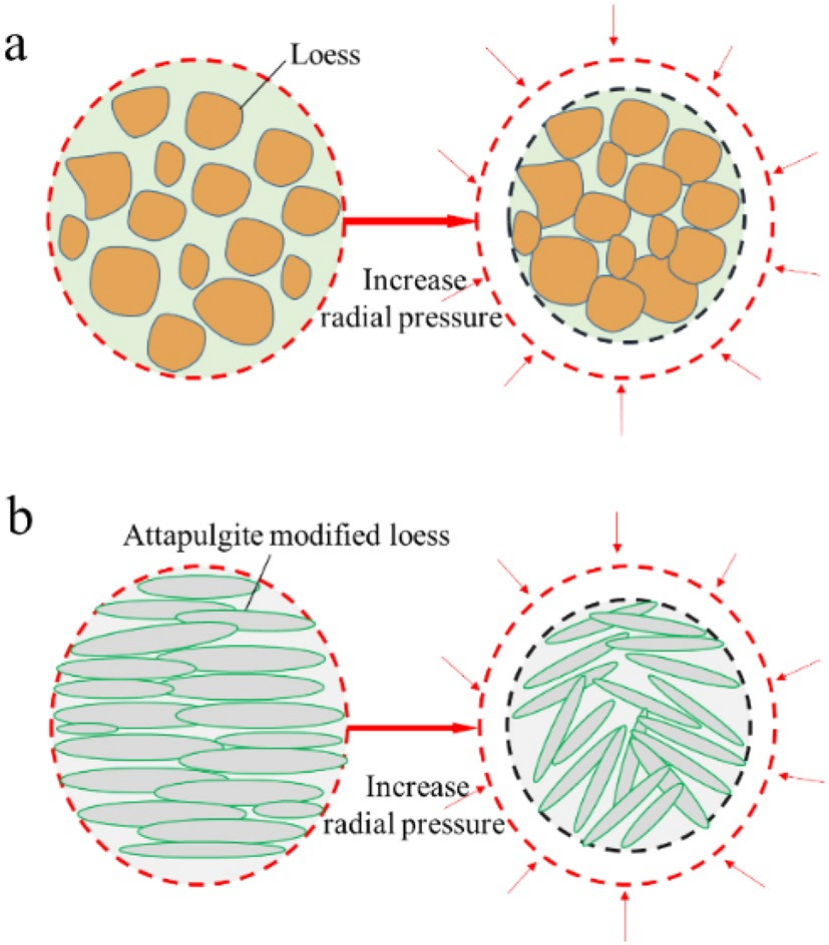
Effect of radial pressure on the pore structure of loess and attapulgite modified loess.

Figure 14 is the schematic diagram of the spatial structure of loess and modified loess specimens changing with the increased radial pressure. There are many pores for the loess specimens in Fig. 14a, and the increase in radial pressure will lead to the redistribution of particles. The gap between particles will decrease. Soil specimens become compacted, and their porosity decreases, leading to a decrease in their permeability coefficient. Attapulgite-modified loess specimens showed lamellar structure formation after loess was combined with attapulgite (Fig. 14b). In its initial state, the lamellar structure was layered and orderly arranged. Compared to loess structure, the pores are smaller, so the permeability coefficient of attapulgite-modified loess was significantly decreased. However, under the same osmotic pressure, the lamellar structure redistribution and the original arrangement are disrupted with the increase of radial pressure. New pores appear in the structure, leading to increased permeability coefficient with increased radial pressure. This aligns with the findings of Guo et al.^39^ and Yang et al.^44^.

## Conclusion

This paper presents the results of various tests, including compaction, penetration, NMR, and SEM, to investigate the permeability of attapulgite-modified loess under different osmotic conditions. The study also observed and analyzed the changes in the microstructure of the specimens before and after infiltration to explain the anti-seepage mechanism of attapulgite-modified loess from a microscopic point of view. The key findings of the research are as follows:

1. As the attapulgite content increases, there is a continuous decrease in the maximum dry density of the modified loess while the optimal water content increases. A predictive equation has been developed to estimate the specimen's maximum dry density and optimal water content for varying levels of attapulgite content.
2. The permeability coefficient of attapulgite-modified loess decreased significantly with an increase in the dosage of attapulgite, but the effect is limited when the content exceeds 10%. The change in the permeability coefficient of attapulgite-modified loess with radial pressure is inversely proportional and directly proportional with the increase in osmotic pressure, albeit to a small extent. Conversely, the permeability coefficient of loess decreases with an increase in radial pressure and increases with an increase in osmotic pressure.
3. The reduction in permeability coefficient with an increase in osmotic pressure may be attributed to the adsorption of water molecules onto the surface of attapulgite particles. This water adsorption could lead to a decrease in the porosity of the specimen and an increase in the tortuosity of the flow path, both of which contribute to a reduction in permeability. In contrast, the increase in permeability coefficient with increasing radial pressure may be because the pressure can cause the inter-particle structure to deform and lead to the formation of new flow paths, which may increase the permeability. The SEM results also support this interpretation, as the sheet structure of the attapulgite-modified loess was observed to change in response to changes in radial pressure, which may lead to the formation of new flow paths and the resulting increase in permeability coefficient.

## Data Availability

The datasets used and/or analysed during the current study available from the corresponding author on reasonable request.
